# Quantitative Optical Diffraction Tomography Imaging of Mouse Platelets

**DOI:** 10.3389/fphys.2020.568087

**Published:** 2020-09-16

**Authors:** Tess A. Stanly, Rakesh Suman, Gulab Fatima Rani, Peter J. O’Toole, Paul M. Kaye, Ian S. Hitchcock

**Affiliations:** ^1^York Biomedical Research Institute, Department of Biology, University of York, York, United Kingdom; ^2^Technology Facility, Department of Biology, University of York, York, United Kingdom; ^3^York Biomedical Research Institute, Hull York Medical School, University of York, York, United Kingdom

**Keywords:** platelets, holotomography, MPN, JAK2V617F, leishmaniasis

## Abstract

Platelets are specialized anucleate cells that play a major role in hemostasis following vessel injury. More recently, platelets have also been implicated in innate immunity and inflammation by directly interacting with immune cells and releasing proinflammatory signals. It is likely therefore that in certain pathologies, such as chronic parasitic infections and myeloid malignancies, platelets can act as mediators for hemostatic and proinflammatory responses. Fortunately, murine platelet function *ex vivo* is highly analogous to human, providing a robust model for functional comparison. However, traditional methods of studying platelet phenotype, function and activation status often rely on using large numbers of whole isolated platelet populations, which severely limits the number and type of assays that can be performed with mouse blood. Here, using cutting edge 3D quantitative phase imaging, holotomography, that uses optical diffraction tomography (ODT), we were able to identify and quantify differences in single unlabeled, live platelets with minimal experimental interference. We analyzed platelets directly isolated from whole blood of mice with either a JAK2V617F-positive myeloproliferative neoplasm (MPN) or *Leishmania donovani* infection. Image analysis of the platelets indicates previously uncharacterized differences in platelet morphology, including altered cell volume and sphericity, as well as changes in biophysical parameters such as refractive index (RI) and dry mass. Together, these data indicate that, by using holotomography, we were able to identify clear disparities in activation status and potential functional ability in disease states compared to control at the level of single platelets.

## Introduction

Maintaining blood flow in basal states and preventing excessive blood loss following injury relies on an orchestrated response from different cell types and non-cellular components, such as clotting factors. Platelets are key cells in this process, becoming rapidly activated following injury and form a platelet plug to reduce blood loss, as well as initiating secondary hemostasis to promote the formation of a stable fibrin-rich thrombus (Machlus and Italiano, 2013). Maintaining a physiological number of functional platelets is essential for hemostasis. However, a wide variety of pathological conditions lead to rapid and sustained changes in platelet counts and functionality. Thrombocytopenia (sustained reduction in platelets of <100 × 10^3^/μL whole blood in humans) is common in autoimmune conditions as well as acute and chronic infections by bacterial, parasitic and viral pathogens including *Salmonella, Staphylococcus aureus, Plasmodium, Leishmania*, and dengue virus (Varma and Naseem, 2010; Cox et al., 2011; Lacerda et al., 2011; Liu et al., 2016; Kullaya et al., 2018; Riswari et al., 2019).

Conversely, thrombocytosis (sustained excess platelet count >450 × 10^3^/μL whole blood in humans) is common in myeloproliferative neoplasms (MPNs), such as essential thrombocythemia (ET). Furthermore, platelet production by megakaryocytes can be significantly altered by bone marrow failure syndromes, whereas inflammatory conditions often impact platelet functionality. In addition to being essential for hemostasis, there is now compelling evidence suggesting key roles for platelets in other processes including wound healing, angiogenesis, inflammation, and innate immunity (Machlus and Italiano, 2013; van der Meijden and Heemskerk, 2019). Platelets express a range of receptors that allow them to interact with and respond to pathogens and assist in regulating an immune response (Assinger, 2014; Hamzeh-Cognasse et al., 2015; van der Meijden and Heemskerk, 2019). There is complex interplay between platelets and bacteria during infections, where the interaction of platelets with immune cells, such as neutrophils generate neutrophil traps/net or release immunomodulatory factors for trapping and clearance of bacteria. Platelets can also bind directly to the bacteria to form clots during inflammatory endocarditis. Platelets assist the formation of the traps, and concurrently the nets contribute to platelet activation, linking inflammation to thrombosis (Gros et al., 2015; Hamzeh-Cognasse et al., 2015).

As platelet production and function is comparable between mice and humans, murine models provide an excellent platform to study the effects of diverse pathologies on platelet form and function. However, traditional methods used for studying platelet phenotype, function and activation status often rely on using large numbers of whole isolated platelet populations. The limited volume of the blood obtained for isolation of platelets severely restricts the number and type of assays that can be performed with mouse blood. Super-resolution imaging techniques have recently enabled the study of structural changes in the protein distribution or cytoskeleton changes of platelets in disease models (Poulter et al., 2015; Bergstrand et al., 2019; Khan and Pike, 2020), However, it is important to note that with improvements in resolution, the post-isolation processing which entails fixation, permeabilization and labeling, can often lead to artifacts if not carefully controlled (Schnell et al., 2012; Stanly et al., 2016; Pereira et al., 2019; Khan and Pike, 2020).

Here, using cutting edge 3D quantitative phase imaging, holotomography, which uses optical diffraction tomography (ODT), we were able to identify and quantify differences in single unlabeled platelets from very small sample volumes. The technique measures 3D differences in the refractive index (RI) tomograms that are generated due to alterations in the diffraction patterns obtained from the cells. This allows the measurement of cellular changes under live conditions, without experimental interferences such as labeling or fixation. With this label-free imaging of the samples we can obtain quantitative information such as cellular dry mass (cytoplasmic concentration) and information on the cell size or structure (Kim et al., 2017; Lee et al., 2019).

Using this technique, we analyzed platelets directly isolated from the whole blood of two different pathological mouse models. In the first model, *Jak/2E/B6 Stella-Cre* mice express a mutated version of human JAK2 (JAK2V617F) under the control of the endogenous *Jak2* promoter (Li et al., 2010). These mice develop symptoms similar to a JAK2V617F-positive MPN, including increased platelets and erythrocytes, megakaryocyte hyperplasia and markers of chronic systemic inflammation. However, the role of JAK2V617F in platelet function remains unclear. *In vitro* analysis of platelets isolated from MPN patients suggests there may be defects in signal transduction and integrin activity (Moore et al., 2013; Lucchesi et al., 2020). While in JAK2V617F-positive mouse models, studies have suggested changes in platelet aggregation *in vitro* (Hobbs et al., 2013) and others identify aberrant hemostasis *in vivo*, but no clear platelet phenotype (Etheridge et al., 2014; Lamrani et al., 2014). Furthermore, as chronic inflammation is now considered a key characteristic of MPNs, the proinflammatory environment may lead to functional changes in platelet activity, leading to the increased incidence of bleeding abnormalities in these patients, as reviewed in Hasselbalch (2012, 2014).

We also analyzed platelets taken from a mouse model of visceral leishmaniasis (VL). In humans, VL causes thrombocytopenia and anemia, a phenotype also mirrored in experimental murine models of *Leishmania donovani*, which is thought to be caused by defective medullary erythropoiesis and thrombocytopenia (Varma and Naseem, 2010; Preham et al., 2018; Rani et al., 2019). The cause of severe thrombocytopenia in the mouse models of VL is unclear, but is likely to be multifactorial, with the parasite infection leading to significant changes in the bone marrow microenvironment and increased macrophage activity that would likely lead to excessive platelet clearance.

In this study, using holotomography, we were able to identify distinct platelet phenotypes in both disease mouse models that may not have been identifiable using current standard assays.

## Materials and Methods

### Ethics Statement

All animal care and experimental procedures were performed under UK Home Office License (Ref # PPL 7008596 and P49487014) and with approval from the Animal Welfare and Ethical Review Board of the Department of Biology, University of York.

### Mice

C57BL/6 (WT) and Jak/2E/B6 Stella-Cre (Li et al., 2010; Kent et al., 2013) (herein referred to as VF) mice were bred at the University of York. For platelet experiments, heterozygous VF mice where used, which exhibit a JAK2V617F+ ET-like phenotype with modest platelet increases, splenomegaly and transformation to myelofibrosis.

For *L. donovani* infection experiments, C57BL/6 female mice at 6–8 weeks of age used for the study. Mice were infected with 3 × 10^7^ amastigotes of the Ethiopian strain of *L. donovani* (LV9) via lateral tail vein, as described (Hasselbalch, 2014). All mice were maintained in individually ventilated cages (at ACDP CL3, where necessary for infection control). All experimental mice were killed 4 weeks post infection.

### Platelet Isolation

The WT and VF mice were euthanized with pentobarbital, followed by collection of whole blood into acid citrate dextrose (ACD) via cardiac punctures. The collected blood was topped up with equal volume of wash buffer (150 mM NaCl, 20 mM HEPES, at RT, pH 6.5) and centrifuged at 60 *g* for 7 min at room temperature without breaks. The platelet rich plasma (PRP) was collected and centrifuged at 240 *g* for 10 min at room temperature to separate platelets and plasma. The platelet pellet was resuspended in wash buffer containing 1 U/ml of apyrase (Sigma-Aldrich, United Kingdom) and 1 μM of prostaglandin E1 (Sigma-Aldrich, United Kingdom) (Prévost et al., 2007).

For *L. donovani* infected mice, whole blood was collected via cardiac puncture after anesthetizing the mice with isoflurane inhalation in a secure chamber (Apollo TEC3 Isoflurane Vaporize, Sound Veterinary Equipment, Rowville, VIC, Australia). Blood was collected in 5 ml polystyrene tubes (Falcon^TM^) coated with ACD and isolated as above into wash buffer without apyrase and prostaglandin E1.

Twenty microliter of the isolated platelets were then added to a TomoDish (Tomocube Inc.) fluidic chamber assembly, where the sample is sandwiched between two #1.5H coverslips.

### Platelet Fixation and Granule Labeling

For monitoring the effect of fixation on platelets, 10 μl isolated platelets in wash buffer were added to the TomoDish with a coverslip and imaged, followed by addition of 10 μl 8% PFA to the flow chamber in the TomoDish allowing it to diffuse in, making a 4% PFA final concentration within the chamber. This was allowed to incubate and imaged every 10 min for a total of 30 min at room temperature (RT).

For labeling the granules, the isolated platelets were incubated, for 30 min at 37°C, 5% CO_2_, with 50 μM Mepacrine (Q3251-25G, Sigma-Aldrich, United Kingdom) to label dense granules or 10 μg/ml of BQ-BSA Green (D12050, Invitrogen, Fisher Scientific) to label the alpha granules. After incubation, the platelets were washed and plated onto the TomoDish with 4% PFA for 10 min at RT. The fixative was removed, and the platelets were then imaged in wash buffer.

### Microscopy

3D Quantitative phase images of platelets were generated using a commercial holotomographic microscope (HT-2H, Tomocube Inc.) that employs ODT using two UPLSAP 60X (NA 1.2) Water dipping lenses (Olympus, Tokyo, Japan). Full details of the optical configuration have been previously described here (Kim et al., 2013; Lim et al., 2015). Samples in wash buffer were mounted on specialized TomoDishes with No1.5H coverslips at RT.

### Statistics

The data were plotted using GraphPad Prism 8 and data analyzed using unpaired or paired two-tailed *t*-tests as indicated in figure legends.

## Results

### 3D Holotomography Image Acquisition and Data Analysis

**FIGURE 1:**
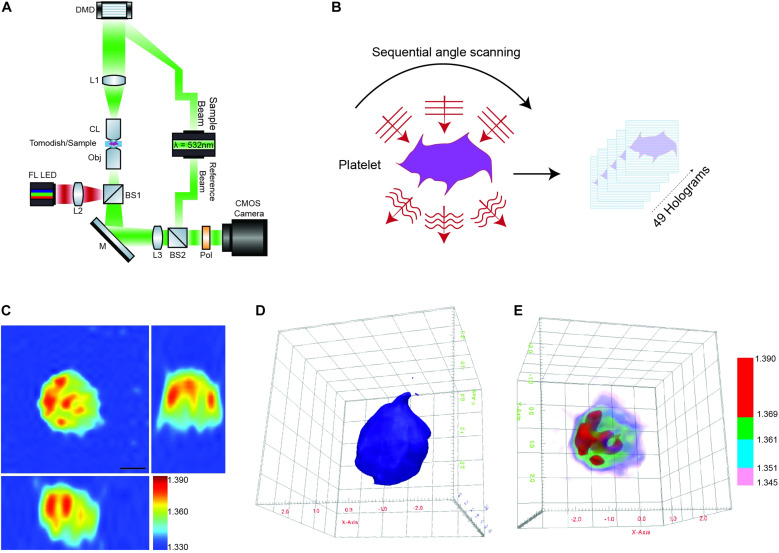
Label-free 3D ODT imaging of platelets. **(A)** Simplified schematic of a holotomographic microscope based on Mach-Zehnder interferometric microscopy. λ-532 nm, laser light source; DMD, digital micromirror device; L1–L3, lenses; CL, condenser lens 60× NA 1.2; Obj, objective lens 60× NA 1.2; M, mirror; BS1-2, beam splitter; Pol, polarizer; FL LED, fluorescence light source. **(B)** Image acquisition/scanning of the sample based on sequential imaging at multiple angles to yield a total of 49 holograms. **(C)** Representative cross-sectional 3D RI tomogram image of a platelet. Color bar represents refractive index. **(D)** Example of 3D surface rendering of the platelet sample used to measure morphological parameters (see also Supplementary Video 1A). **(E)** Representative RI based rendering, where the pseudo coloring represents different bands of refractive index to highlight intracellular features of the platelet (see also Supplementary Video 1B).

Briefly, the HT-2H microscope is based on a Mach–Zehnder interferometer equipped with a digital micromirror device (DMD) (Figure 1A). Using a coherent monochromatic laser (λ = 532 nm) divided into a sample and reference beam, 2D holographic QPI images were generated at multiple illumination angles (Figure 1B) where the incidence light is accurately controlled by the DMD (Shin et al., 2015). The light beam diffracted by the sample was collected using a high numerical aperture (NA = 1.2) objective lens UPLSAP 60XW (Olympus, Tokyo, Japan), with the subsequent holograms recorded on a CMOS sensor. A 3D RI tomogram was then reconstructed from the series of off-axis holograms using the TomoStudio^TM^ software (Tomocube Inc.) (Figure 1C). Further details of the algorithms used and principles of ODT can be found here (Shin et al., 2016).

The 3D RI Tomograms of individual platelets were visualized and segmented to measure three-dimensional morphological parameters, mean RI and dry mass within the TomoStudio^TM^ software (Figures 1D,E and Supplementary Videos 1A,B).

### Platelets From Myeloid Malignancies Show Changes in Platelet Morphologies and Intracellular Components

**FIGURE 2:**
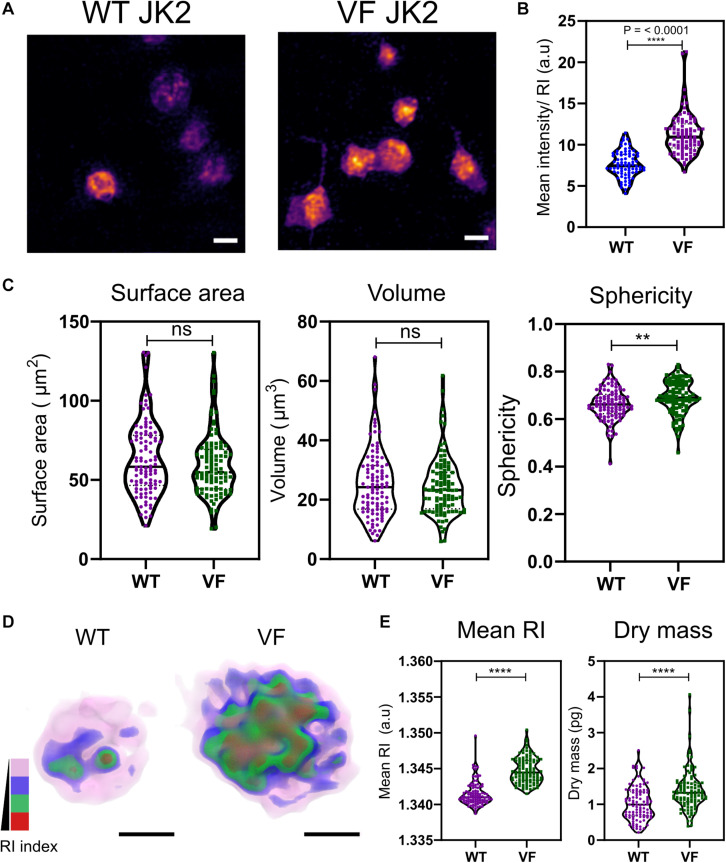
Morphological and biophysical differences in platelets from WT and JAK2V617F+ (VF) from murine blood. **(A)** Max RI projections of the 3D phase images of platelets from WT and VF mice. **(B)** The mean intensity values from the RI projections between WT and VF platelets (unpaired two-tailed *t*-test, *P* ≤ 0.0001, *n* = 80 platelets). **(C)** Morphological changes of platelets from WT and VF mice in surface area platelets (unpaired two-tailed *t*-test, *P* = 0.1921), volume (unpaired two-tailed *t*-test, *P* = 0.5062), and sphericity (unpaired two-tailed *t*-test, *P* = 0.0063 *n* = 102 platelets, median represented by black line in the violin plot. Scale bar 2 μm. **(D)** 3D rendered iso-surface image of platelets from WT and VF mice based on RI. **(E)** Changes in mean RI (unpaired two-tailed *t*-test, *P* ≤ 0.0001) and dry mass (unpaired two-tailed *t*-test, *P* ≤ 0.0001) of the platelets. Scale bar 1 μm, RI scales; Pink: 1.3426–1.3483, blue: 1.3484–1.3519, green: 1.3520–1.3565, red: 1.3566–1.3628. **P ≤ 0.01, ****P ≤ 0.0001.

Aberrant hemostasis and thrombosis is one of the most common causes of morbidity and mortality in JAK2V617F-positive MPN patients. Although some differences have been identified in platelet protein expression levels that would lead to a prothrombotic phenotype, the actual structure or morphology of these platelets has yet not been visualized. In order to study the differences in these platelets from WT vs. VF mice, we used a previously characterized murine model system for JAK2V617F+ mice (Li et al., 2010). Maximum RI projection images of unfixed platelets, freshly isolated from WT and VF mice indicated no visible change in the morphology (Figure 2A). However, quantitative analysis of the RI projection data indicated that had higher intensity compared to WT platelets (Figure 2B; *P* < 0.001), suggesting differences in composition between platelets from these two strains. There were no significant differences between WT and VF platelets in surface area or volume, but VF platelets had a significant increase in sphericity (unpaired two-tailed *t*-test, *P* = 0.1921, *P* = 0.5062, and *P* = 0.0063, respectively; Figure 2C). Thus, platelets in VF mice are altered in shape but not size.

We next created 3D iso-surface rendered images of the platelets based on a range of RIs, to provide data about cytosolic differences between cells (Kim et al., 2017; Lee et al., 2019). The 3D iso-surface rendered images of the platelets from WT and JAK2V617F mice show a clear difference in the organization of RI materials within the platelets (Figure 2D). corresponding with the RI values (Figure 2E; unpaired two-tailed *t*-test, *P* ≤ 0.0001) and a significant increase in the dry mass (unpaired two-tailed *t*-test, *P* ≤ 0.0001).

Bioactive molecules, such as those stored in alpha granules [containing membrane associated proteins – integrins, P-selectin, and soluble factors – vWF, factor V to name a few (Blair and Flaumenhaft, 2009; Flaumenhaft and Sharda, 2018)] and dense granules [mainly contain calcium, serotonin, histamines, etc., (Flaumenhaft and Sharda, 2018)], are key components within the platelets that make them active and respond to specific agonists (Hamzeh-Cognasse et al., 2015). In order to identify these granules and map the RI, we labeled the granules using membrane permeable dyes (Hanby et al., 2017) and fixed the platelets with 4% PFA for 10 min. The PFA fixation at 10 min does not affect the size, shape, RI and dry mass of the platelets but leaving them from longer than 10 min significantly alters these parameters (Supplementary Figures S1A–E). Unfortunately, although we could detect alpha and dense granules within the platelets, we were unable to measure the exact RI for the granules (Supplementary Figures S1F,G).

### *L. donovani* Infected Mice Have Altered Platelet Morphologies and Intracellular Features

To determine whether similar changes occurred in another pathological setting characterized by alterations in platelet numbers, we examined platelets from mice with experimental VL (Hasselbalch, 2014; Preham et al., 2018).

**FIGURE 3:**
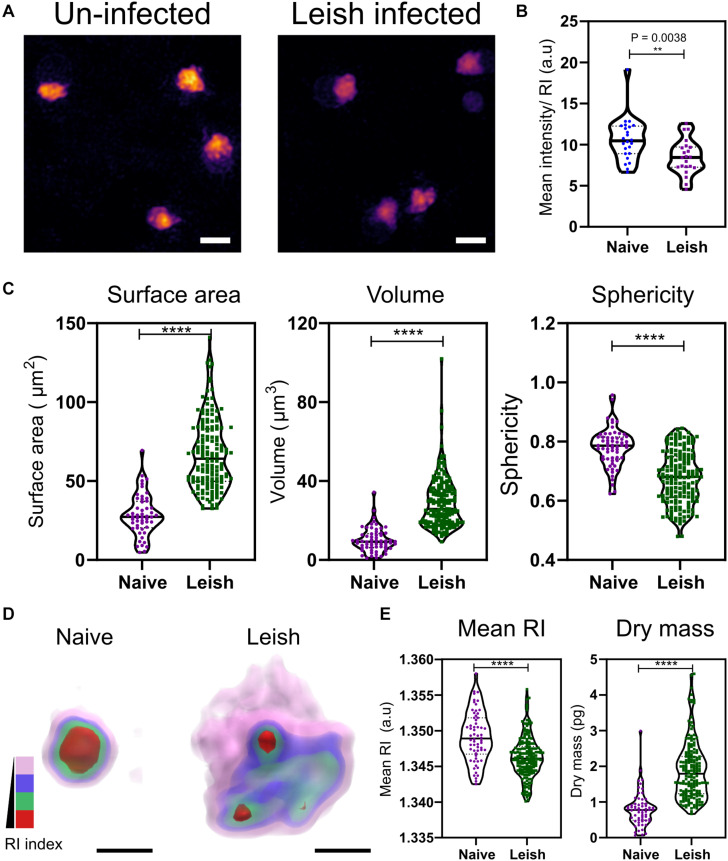
Morphological and biophysical differences in platelets from naïve and Leishmania infected mice. **(A,B)** Max RI projections of the 3D phase images of platelets from Naïve and *L. donovani* infected mice *n* = 23 platelets. **(C)** Morphological changes seen in platelets in surface area, volume, and sphericity. *n* = 62 platelets, 10 platelets from each WT mice in study (6 mice) and *n* = 172 platelets from 17 *L. donovani* infected mice, median represented by black line in the violin plot. Scale bar 2 μm. **(D)** 3D rendered iso-surface image of platelets from naïve and *L. donovani* infected mice based on RI. Scale bar 1 μm. **(E)** Changes in mean RI (unpaired two-tailed *t*-test, *P* ≤ 0.0001), and dry mass (unpaired two-tailed *t*-test, *P* ≤ 0.0001) of the platelets. RI index scales; Pink: 1.3406–1.3525, blue: 1.3525–1.3621, green: 1.3622–1.3735, red: 1.3736–1.3822. **P ≤ 0.01, ****P ≤ 0.0001.

Visible changes in the morphology of the platelets was observed in maximum RI projection images of the platelets freshly isolated from naïve and *L. donovani*-infected WT C57BL/6 mice (Figure 3A). In contrast to VF mice (Figure 2), platelets from *L. donovani*-infected mice have a much lower mean intensity (unpaired two-tailed *t*-test, *P* = 0.0038; Figure 3B). Platelets from infected mice had increased surface area and volume but reduced sphericity (unpaired two-tailed *t*-test all *P* ≤ 0.0001; Figure 3C) Thus, platelets from infected mice had an increase in size as well as an altered intracellular environment and a suggestion, based on sphericity, of activation.

Similar to the VF model, we also studied changes in the cytosolic parameters. Platelets from naïve and *L. donovani*-infected mice also showed a visible difference in the RI (Figure 3D), with a significant reduction in higher RI based structures (unpaired two-tailed *t*-test, *P* ≤ 0.0001; Figure 3E). Unlike VF platelets, platelets from *L. donovani*-infected mice had an increase in dry mass (unpaired two-tailed *t*-test, *P* ≤ 0.0001; Figure 3E).

## Discussion

Imaging based techniques are rising in popularity with the advancement of super-resolution imaging and the amount of quantitative information concerning the functioning of the cells that can be derived from such experiments. The field of platelet biology has taken advantage of these tools and a vast array of new and exciting information relating to the changes in the internal structure and function of platelets or platelet proteins during activation processes or disorders has recently been produced (Poulter et al., 2015; Westmoreland et al., 2016; Knight et al., 2017; Bergstrand et al., 2019; Khan and Pike, 2020). With fluorescence based imaging, the protein of interest often requires the samples to have a fluorescent tag – either genetically modified or by immuno-labeling, to provide the contrast required for visualization. In our study, we use 3D holotomography, a RI-based imaging technique, that requires no labeling or post-isolation processing, enabling us to study alterations in the cell morphology and changes in biophysical parameter of unaltered cells or platelets in health and disease.

Obtaining enough platelets from mice to perform standard *in vitro* functional assays, such as lumi-aggregometry or western blotting, has always been significant restraint for platelet studies. As a result, researchers are often limited to a single assay for each mouse. However, the assays outlined here can be performed with a minimum volume of 10–20 μl/chamber, allowing sampling of 100–200 platelets at single cell level, sufficient to generate quantitative information relating to platelet morphology and RI/dry mass. Therefore, this imaging technique can serve as a valuable additional or complementary technique to other *in vitro* platelet function assays.

Here, we used this technique to study the morphological changes and alterations in the cellular composition of platelets directly isolated from two murine disease models. We isolated and imaged platelets from mice with either somatic mutations in JAK2, JAK2V617F, that cause MPNs or platelets from mice with experimental VL following infection with *L. donovani*. Both of these models have characteristic alterations in their platelets, leading to either thrombocytosis or thrombocytopenia with chronic inflammation giving rise to these fatal thrombotic or bleeding disorders (Goncalves et al., 2011; Hasselbalch, 2012, 2014; Etheridge et al., 2014).

Under normal physiological conditions, the vascular endothelium continuously suppresses platelet activation via expression of ectonucleotidases, thrombomodulin, or by releasing prostaglandin I2 and nitric oxide. This is usually altered when there is an inflammatory environment or vascular injury that contributes to a prothrombotic phenotype, as seen in bleeding disorders (van der Meijden and Heemskerk, 2019). We have previously shown, using an alternative murine JAK2V617F-positive MPN model, that expression of the mutated protein in platelets did not have a significant effect on aggregation *in vitro*, and aberrant hemostasis was largely due to extreme thrombocytosis-induced acquired von Willebrand’s disease and an inflamed endothelial environment (Etheridge et al., 2014). An inflamed endothelium is common in MPNs due to a chronic inflammatory environment and often leads to atherosclerosis and secondary cancers (Hasselbalch, 2012, 2014). This inflammatory environment is characterized by increases in circulating thrombomodulin, selectins and von Willebrand factor. This is turn activates the endothelial cells lining the blood vessels, leukocytes and platelets (Hasselbalch, 2014). Thus the platelets have a significant role to play in the MPN bleeding complications, often with progression to secondary complications (Hasselbalch, 2012; Etheridge et al., 2014). In our study, platelets isolated from VF mice do not have a characteristic change in morphology, but do show an increase in the mean RI and dry mass (Figure 2) indicating intracellular changes within the platelets, which may largely be due to platelets being primed by the inflammatory environment for further activation and function.

The larger the size of the platelets, the greater is reactivity and prothrombotic ability (Slavka et al., 2011; Machlus and Italiano, 2013). Platelets from *L. donovani* infected C57BL/6 mice were found to be much larger in size and less spherical compared to the naïve platelets (Figure 3), presenting a much more morphologically activated state, accompanied by changes in the intracellular content. The larger platelets were also seen in H&E staining of blood smears from infected mice (Rani et al., 2019 and Rani G et al., unpublished). The mechanism(s) underlying these changes in platelets during infection remain to be determined. The inflammatory response to *L. major* infection has previously been associated with platelet activation, release of platelet derived growth factor (PDGF) and amplification of monocyte recruitment via CCL-2 (Goncalves et al., 2011). Platelet activation is also known to alter sialylation of surface glycoproteins allowing recruitment to the inflamed liver and clearance by hepatocytes via Ashwell-Morrell Receptor (Rani et al., 2019 and Rani G et al., unpublished). Furthermore, an increase in the number of IgG-bound platelets in *L. donovani* infected mice likely underpins enhanced clearance of activated platelets, leading to severe thrombocytopenia in these models. The presence of larger platelets, as seen in our results, could also be due to newly formed platelets owing to increased turnover and changes in platelet biogenesis [Rani et al. (unpublished); and Slavka et al. (2011)].

In both models studied, we noticed a different platelet RI for the WT and naïve mice and a non-linear relationship of RI and dry mass between naïve and *L. donovani* infected platelets (Figures 2, 3). We currently do not know the effect of the different platelet isolation methods and buffers (see “Materials and Methods”) have on the RI of the platelets. We speculate this to be one of the reasons resulting in the RI difference. Day to day calibration of the system based on the “buffer background” is done prior to imaging which could also cause these minor alterations in the resulting RI between the two models.

Based on ODT literature, RI and dry mass are linearly proportional (Kim et al., 2016, 2017; Lee et al., 2019). In the *L. donovani* infection model, this does not seem to be the case. The naïve platelets, in the infection model, show a broad distribution of RI data sets – some with higher RI and others lower. The corresponding dry mass shows a tighter distribution of the data set. This difference is noted in both models, with the WT mice having a tighter RI range and a broader dry mass in contrast to the naïve platelets. But strangely, this occurs only in the WT and naïve platelets, while the VF and *L. donovani* infected platelets do not show this pattern. Human platelets isolated from different donors have been shown to have some variation in their morphological and cytosolic parameters and also have altered activation patterns when stimulated (Lee et al., 2019). Therefore, the differences in our results may be an inherent property of the normal, uninfected platelets that may have gone unnoticed with other platelet assays. Thus, from these two differences highlighted in our results, we can only compare and contrast RI data from within one experimental data.

Both mouse models used in our study share several hematological pathologies such as splenomegaly, largely associated with thrombocytopenia in leishmaniasis, and an inflammatory environment (Hasselbalch, 2012; Sangkhae et al., 2015; Preham et al., 2018). This inflammatory environment causes an influx of immune cell responses, where platelets can act as mediators for generating these responses (Garraud and Cognasse, 2015; Gros et al., 2015). The role of platelets in innate immunity is largely due to their capacity to internalize viruses and bacteria, release of bioactive molecules stored in their granules and interaction with other cells, i.e., leukocytes to combat a potential threat (Li et al., 2012; Ali et al., 2015). Overall, the complex nature of platelet function as either pro- or anti- inflammatory, or the platelets having a beneficial or detrimental effect is entirely dependent on the cause of the inflammation and the pathophysiology of the disease as described in the review by Gros et al. (2015). Here, using our label-free method of RI imaging, we were able to highlight that platelets isolated from our hematological and inflammatory disease models appear different in structure and size and cytosolic concentrations, compared to WT controls. Although there are some limitations in using the fluorescence-based imaging with this platform, there is more RI based information and real-time cell dynamics that can be tracked over time (Lee et al., 2019), allowing this method to be used for studying the effect of different drugs on the activation states of platelets. The technique also highlights the amount of valuable information that can be derived from only a fraction of the platelets isolated from mice, which will aid in complementing other functional assays of platelet activation.

## Data Availability Statement

All datasets presented in this study are included in the article/Supplementary Material.

## Ethics Statement

The animal study was reviewed and approved by the UK Home Office Animal Welfare and Ethical Review Board of the Department of Biology, University of York.

## Author Contributions

TS contributed to the experimental design, experimental work, imaging, data analysis, and manuscript preparation. RS contributed to the imaging and data analysis. GR contributed to the experimental work and manuscript preparation. PO’T contributed to the experimental work. PK contributed to the *L. donovani* project supervision and manuscript preparation. IH contributed to the project supervision, experimental design, and manuscript preparation. All authors contributed to the article and approved the submitted version.

## Conflict of Interest

The authors declare that the research was conducted in the absence of any commercial or financial relationships that could be construed as a potential conflict of interest.

## Supplementary Material

FIGURE S1 Four percentage PFA fixation on platelets at varying time scales. **(A–E)** Morphological and biochemical parameters affected by PFA fixation **(A)** Surface area (*P* = 0.3263, 0.0010, 0.0083), **(B)** Volume (*P* = 0.4913, <0.0001, 0.0004), **(C)** Sphericity (*P* = 0.1521, 0.0027, 0.0103), **(D)** RI (*P* = 0.4214, 0.9495, 0.6858), and **(E)** Dry mass (*P* = 0.7976, 0.0005, 0.0001). Paired two tailed *t*-test was performed for all, *n* = 30. RI: Pink: 1.3444–1.3525, blue: 1.3526–1.3621, brown: 1.3622–1.3719, red: 1.3720–1.3775.
