# Supplementary figures and images for: Quantitative Optical Diffraction Tomography Imaging of Mouse Platelets
Source: Front Physiol. 2020 Sep 16;11:568087. doi: 10.3389/fphys.2020.568087 (PMC7526686; doi:10.3389/fphys.2020.568087)

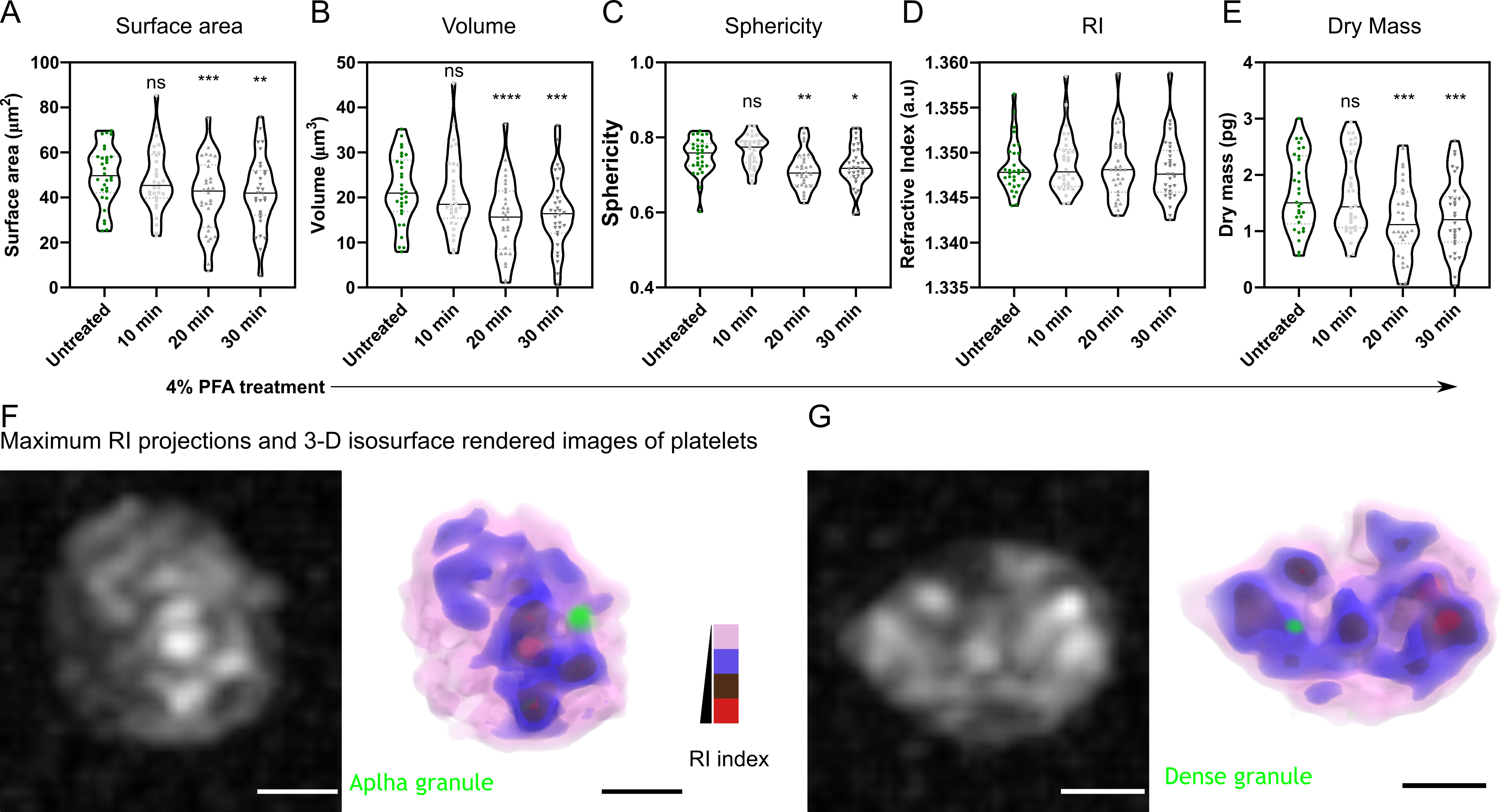

Supplement: FIGURE S1 — Four percentage PFA fixation on platelets at varying time scales. (A–E) Morphological and biochemical parameters affected by PFA fixation (A) Surface area (P = 0.3263, 0.0010, 0.0083), (B) Volume (P = 0.4913, <0.0001, 0.0004), (C) Sphericity (P = 0.1521, 0.0027, 0.0103), (D) RI (P = 0.4214, 0.9495, 0.6858), and (E) Dry mass (P = 0.7976, 0.0005, 0.0001). Paired two tailed t-test was performed for all, n = 30. RI: Pink: 1.3444–1.3525, blue: 1.3526–1.3621, brown: 1.3622–1.3719, red: 1.3720–1.3775. [file Image_1.TIF]
